# Case Report: Sengers syndrome caused by a novel 7.6 kb AGK deletion misdiagnosed as isolated congenital cataract

**DOI:** 10.3389/fped.2026.1714952

**Published:** 2026-01-30

**Authors:** Xingwang Gong, Yue Liu, Hui Liang

**Affiliations:** 1 Department of Neurology and Institute of Neurology of First Affiliated Hospital, Institute of Neuroscience, and Fujian Key Laboratory of Molecular Neurology, Fujian Medical University, Fuzhou, China; 2 Department of Neurology, Hainan General Hospital, Hainan Affiliated Hospital of Hainan Medical University, Hainan Clinical Medical Center, Hainan Academician Team Innovation Center, Haikou, China

**Keywords:** *AGK*, breakpoint mapping, case report, copy number variation, genomic deletion, Sengers syndrome

## Abstract

The diagnosis of Sengers syndrome, a rare mitochondrial disorder, is often challenged by phenotypic mimicry. We report a diagnostically instructive case of a 4-month-old female who presented with the classic triad of congenital cataracts, hypertrophic cardiomyopathy, and lactic acidosis. Initial whole-exome sequencing (WES) was confounded by the finding of a heterozygous variant in *CRYBA2* and only a single heterozygous nonsense mutation in *AGK* (c.409C>T, p.Arg137*). The persistence of a multisystemic phenotype inconsistent with an isolated cataract disorder prompted further investigation. Copy number variation (CNV) analysis of the WES data revealed a large heterozygous deletion in *AGK*, which breakpoint-specific polymerase chain reaction and Sanger sequencing precisely characterized as a novel 7.6 kb deletion (chr7:141297542-141305156). This confirmed compound heterozygosity, yielding a definitive diagnosis of Sengers syndrome and reclassifying the *CRYBA2* variant as incidental. Crucially, breakpoint analysis indicated a non-Alu-mediated mechanism for the deletion. This case highlights the critical importance of CNV analysis in resolving genetically ambiguous autosomal recessive cases and provides novel insight into the structural mutational landscape of *AGK*.

## Introduction

**Figure 1:**
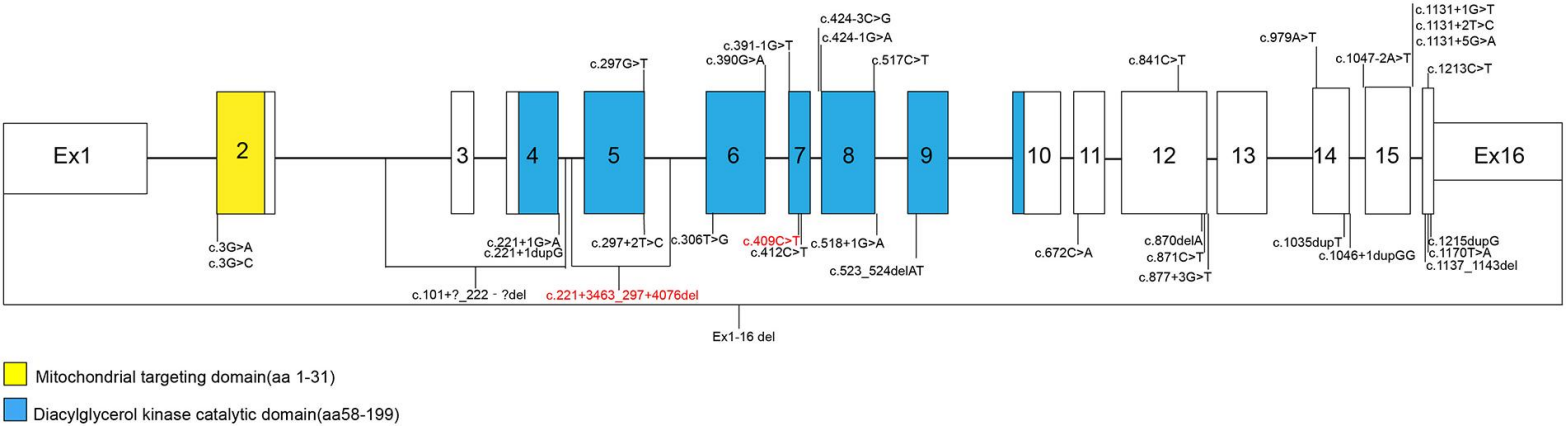
Schematic of the *AGK* gene with reported pathogenic variants and the novel deletion identified in this study. The structure of the *AGK* gene (NM_018238.4) is depicted, with exons shown as numbered boxes and introns as connecting lines. The positions of previously reported pathogenic mutations are indicated above the schematic. The compound heterozygous mutations identified in our patient are highlighted in red: the maternally inherited nonsense mutation c.409C>T (p.Arg137*) and the novel, paternally inherited 7.6 kb genomic deletion (chr7:141297542-141305156) encompassing exon 5.

Sengers syndrome is a rare autosomal recessive mitochondrial disorder characterized by congenital cataracts, hypertrophic cardiomyopathy, and lactic acidosis (1, 2). A significant diagnostic challenge arises because the early and prominent cataracts can misleadingly steer genetic investigation towards genes associated with isolated ocular disease, such as *CRYBA2* (3). The discovery of a heterozygous variant in these genes represents a classic diagnostic pitfall. Since its link to biallelic *AGK* mutations was established, numerous pathogenic variants have been reported in the literature (4–8). As of July 2025, a combined query of the ClinVar and LOVD databases identified 34 unique pathogenic or likely pathogenic variants in *AGK* (Figure 1). However, large genomic rearrangements in *AGK* remain underreported, partly because conventional sequencing often lacks dedicated copy number variation (CNV) interrogation. Moreover, the precise architecture and formation mechanisms of such deletions are seldom investigated.

We present an instructive case that addresses these diagnostic and mechanistic gaps. In a patient with the classic clinical triad, initial whole-exome sequencing (WES) was confounded by a heterozygous *CRYBA2* variant and only a single heterozygous *AGK* nonsense mutation. This case is unique because: (1) it exemplifies the resolution of a diagnostic dilemma through persistent CNV analysis, leading to the identification of a novel *AGK* deletion, and (2) it leverages precise breakpoint mapping to provide evidence for a non-Alu-mediated deletion mechanism, which differs from the Alu-mediated recombination model commonly observed in many genomic disorders. This report thereby highlights both the clinical necessity of comprehensive genetic analysis and its value in advancing molecular pathogenesis insights.

## Case report

**Figure 2:**
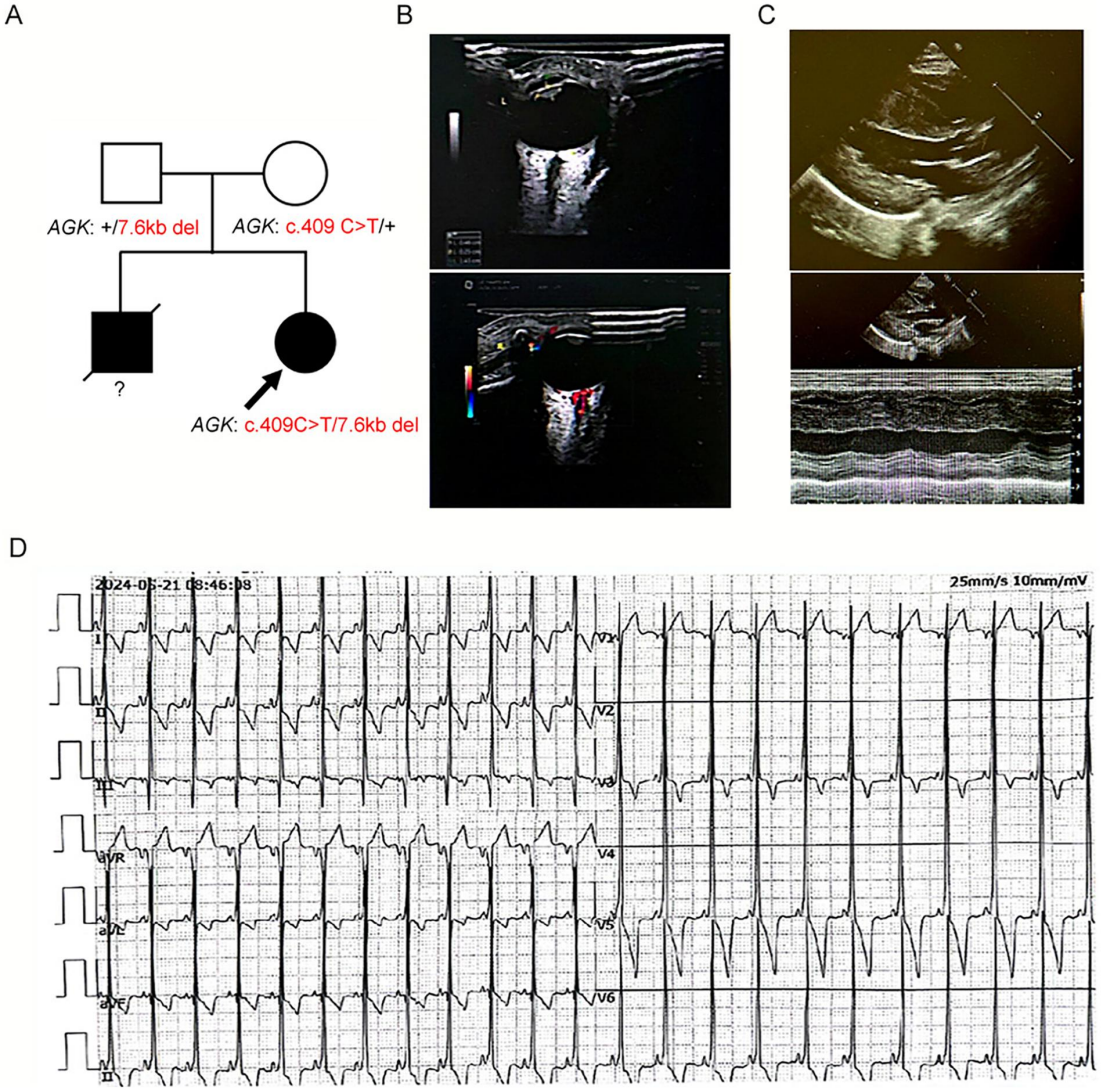
Diagnostic workup of the index case. **(A)** Family pedigree. The proband (indicated by arrow) is the second child of non-consanguineous parents. The family history is notable for a deceased male sibling. Squares represent males, circles females; filled symbols indicate affected individuals. **(B)** Ocular ultrasound showing hyperechoic signals within the lens, confirming congenital cataracts. **(C)** Echocardiogram demonstrating pronounced hypertrophy of the interventricular septum and left ventricular wall, consistent with hypertrophic cardiomyopathy. **(D)** Electrocardiogram tracing revealing a complex arrhythmia profile, including sinus rhythm with ventricular preexcitation, supraventricular premature contractions, and nonspecific ST-T segment changes.

In March 2022, a 4-month-old female infant was admitted to our tertiary care center for evaluation of bilateral congenital cataracts, cardiac dysfunction, and persistent metabolic acidosis. She was the product of a non-consanguineous marriage, with a family history notable for the unexplained death of a male sibling shortly after birth (Figure 2A). Physical examination upon admission revealed diminished visual responsiveness, cardiomegaly on percussion, and generalized muscular hypotonia. The diagnostic workup confirmed the clinical suspicions: ocular ultrasound identified hyperechoic signals within the lens, consistent with congenital cataracts (Figure 2B); echocardiography demonstrated pronounced hypertrophic cardiomyopathy with impaired systolic function (left ventricular ejection fraction, 45%; Figure 2C); electrocardiography revealed a complex arrhythmia profile, including sinus rhythm with ventricular preexcitation, supraventricular premature contractions, and nonspecific ST-T segment changes (Figure 2D); and laboratory investigations revealed severe lactic acidosis (blood lactate level, 9.8 mmol/L). The constellation of findings was highly indicative of Sengers syndrome.

The timeline of her care is summarized in Table 1. Key milestones included the postnatal diagnosis of cataracts, the emergence of cardiac and metabolic symptoms around three months of age leading to referral, and the comprehensive inpatient evaluation at four months that solidified the clinical diagnosis.

**Table 1:** Timeline of clinical care and diagnostic milestones.

Time point/age	Clinical event or intervention	Outcome/Finding	Clinical decision/Action
Birth	Neonatal diagnosis of bilateral cataracts	Referral to ophthalmology	Discussed future cataract surgery
3 months	Onset of feeding difficulties, lethargy, FTT	Concern for systemic disorder	Urgent referral to tertiary center
4 months (Admission)	Suspected Sengers syndrome	Cataracts, HCM (EF45%),arrhythmia, lactic acidosis (9.8 mmol/L)	WES initiated. Discharged with palliative care plan pending results
5–6 months (Follow-up)	Initial WES report received	Heterozygous AGK & CRYBA2 variants (inconclusive)	CNV analysis requested on existing WES data
6–7 months	CNV analysis & validation completed	Novel paternal AGK deletion confirmed	Definitive molecular diagnosis of Sengers syndrome established
After Diagnosis	Genetic Counseling provided	Explained diagnosis, poor prognosis, recurrence risk. Clinical course was marked by progressive deterioration	Focus on palliative support

FTT, failure to thrive; HCM, hypertrophic cardiomyopathy; EF, ejection fraction; ECG, electrocardiography; WES, whole exome sequencing; CNV, copy number variation; AGK, Acylglycerol Kinase.

**Figure 3:**
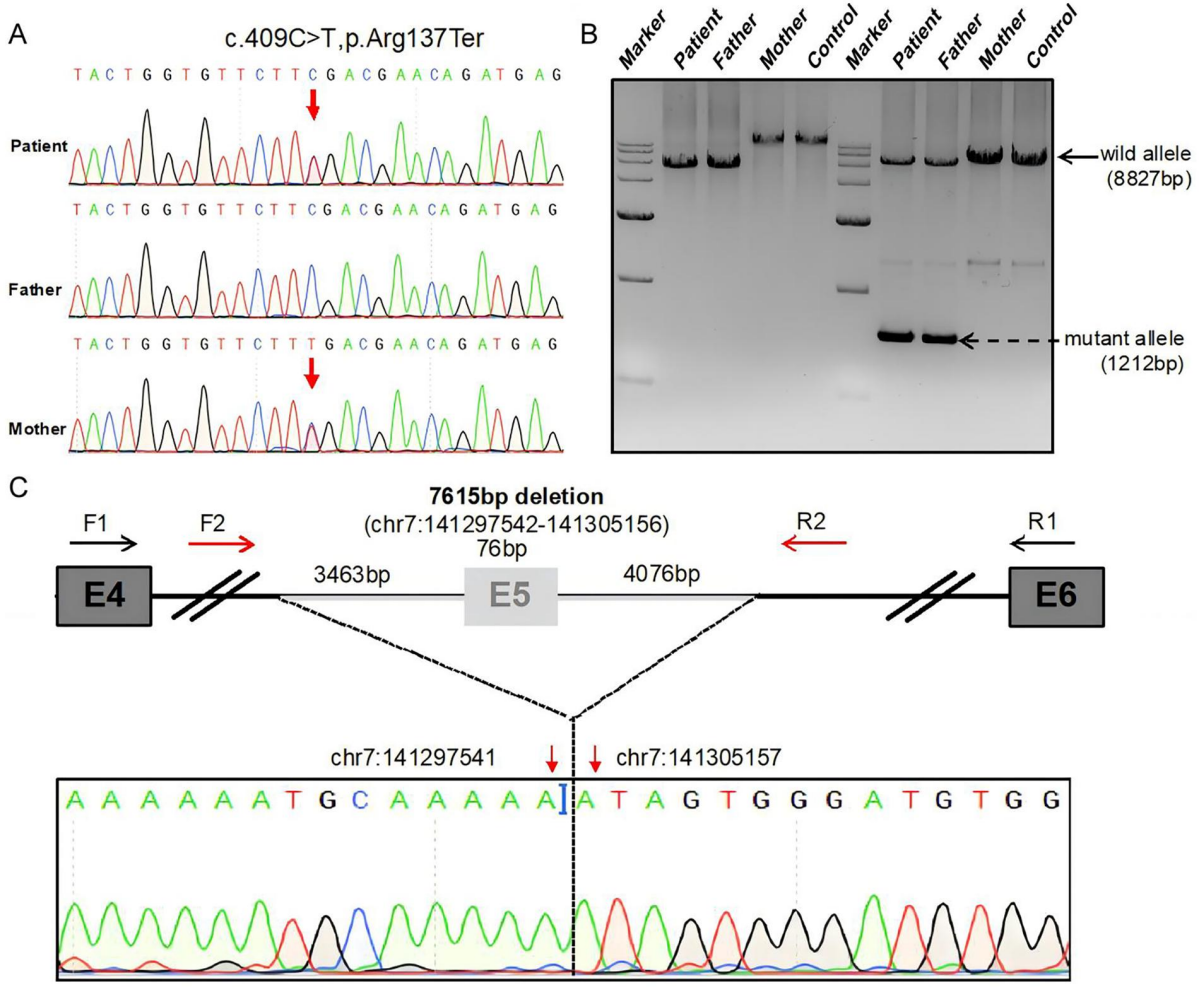
Identification of a novel *AGK* deletion via sanger sequencing and PCR analysis. **(A)** Sanger sequencing confirmed the heterozygous c.409C>T mutation in the proband and her mother. The father carried the wild-type sequence at this position. **(B)** PCR analysis of the genomic deletion. (Left) Initial long-range PCR yielded a shorter product in the proband and her father, suggesting a deletion. (Right) A subsequent PCR assay with flanking primers produced two amplicons: an 8,827 bp fragment (wild-type allele) and a 1,212 bp fragment (deletion allele). The proband and her father exhibited both bands, while the mother and a control subject showed only the 8,827 bp band. **(C)** Sanger sequencing of the purified 1,212 bp band from the proband and father validated the breakpoints of the heterozygous deletion (chr7:141297542–141305156).

To confirm the molecular diagnosis, WES was performed. Initial analysis identified two heterozygous variants: a pathogenic nonsense variant in *AGK* (GRCh37/hg19: chr7: g.141313964 C>T; NM_018238.4: c.409C>T; p.Arg137*), as confirmed by Sanger sequencing (Figure 3A), which was classified as Pathogenic according to the guidelines of the American College of Medical Genetics and Genomics and the Association for Molecular Pathology (ACMG/AMP) (criteria: PVS1, PS1, PM2, PP3) and was maternally inherited; and a missense variant in *CRYBA2* (GRCh37/hg19: chr2: g.219855111 A>G; NM_057093.2: c.457T>C; p.Tyr153His), shown by segregation analysis to be paternally inherited. We formally applied the ACMG/AMP framework to the *CRYBA2* variant. It was classified as a Variant of Uncertain Significance, with the assessment primarily anchored in criterion BS4 (lack of segregation with disease): the variant was present in the proband's healthy, asymptomatic father, who displayed no features of congenital cataract or any systemic manifestations of Sengers syndrome. Although the variant's exceptionally low population frequency (gnomAD overall: 6.196e-7; absent in East Asian subsets) provided nominal support for pathogenicity (PM2), the direct clinical evidence from the family (BS4) carried greater diagnostic weight in this context. Moreover, a heterozygous *CRYBA2* variant is incompatible with the patient's multisystemic presentation. Crucially, the full clinical phenotype was conclusively explained by the biallelic pathogenic variants in *AGK*. Therefore, the *CRYBA2* p.Tyr153His variant was deemed an incidental finding, a clear example of how a phenotypically salient variant can misleadingly steer initial interpretation. This conclusion necessitated further investigation to identify the second pathogenic allele in *AGK*.

To detect CNV from the WES data, we performed CNV analysis using EXCAVATOR2, a dedicated algorithm for exon-level CNV calling from exome sequencing data. A minimum exon coverage of 30× and a log_2_ ratio threshold of ±0.8 were applied to identify heterozygous deletions. This finding was validated and characterized using breakpoint-specific polymerase chain reaction (PCR) and Sanger sequencing. PCR analysis confirmed a heterozygous deletion in the proband and her father (Figure 3B). Subsequent Sanger sequencing of the deletion-specific amplicon precisely mapped the novel, paternally inherited 7.6 kb deletion (chr7:141297542-141305156) encompassing exon 5 of *AGK* (Figure 3C). Analysis of the deletion breakpoints revealed no significant homology or flanking Alu repeats, suggesting a non-Alu-mediated mechanism underlying this rearrangement. Segregation analysis confirmed the compound heterozygous state in the proband, leading to a definitive molecular diagnosis of Sengers syndrome (Figure 2A).

Following the diagnosis, a multidisciplinary palliative care plan was initiated. The rapid progression of her cardiac disease precluded surgical intervention for cataracts, necessitating a focus on intensive medical management of her cardiomyopathy and metabolic acidosis. The patient was discharged with home-based palliative support, and subsequent follow-up indicated that her clinical course was marked by progressive deterioration. The family received comprehensive genetic counseling, and written informed consent for publication was obtained from the parents.

## Discussion

This report delineates the diagnostic odyssey of an infant with Sengers syndrome, ultimately resolved by identifying a novel compound heterozygous genotype in *AGK*. Our case underscores a recurrent pitfall in clinical genetics, where an incidental finding can obscure a systemic diagnosis, and highlights the critical role of CNV analysis in closing such diagnostic gaps. Furthermore, the precise characterization of the deletion breakpoint offers a novel perspective on the mutational mechanisms operative at the *AGK* locus.

Our diagnostic approach involved an iterative and comprehensive genetic investigation. Confronted with a phenotype-genotype mismatch after initial WES, we extended the analysis to interrogate the existing data for structural variants, a step that proved decisive. This persistence underscores that a single heterozygous finding in an autosomal recessive disorder should not terminate the diagnostic inquiry but rather prompt a search for a second, potentially cryptic allele. The subsequent molecular characterization of the deletion, providing mechanistic insight beyond mere detection, represents a further strength. The main limitation of this study is the absence of functional validation in patient-derived cells, which could have directly demonstrated the impact of the deletion on *AGK* expression and mitochondrial function. However, the nature of our report as a single observation is not a limitation per se, but rather a characteristic of case reports, whose value lies in describing novel findings that challenge existing models and generate new hypotheses. The pathogenicity of the identified deletion is strongly supported by its predicted null effect and perfect segregation with the disease.

Our experience resonates with the documented challenges of diagnosing rare mitochondrial disorders. The potential for a striking feature like congenital cataracts to misleadingly implicate genes associated with isolated anomalies is a well-known diagnostic trap. The resolution of our case through CNV analysis aligns with a growing literature emphasizing its indispensability in modern genetic testing (9). While over 30 pathogenic *AGK* variants have been reported, large genomic rearrangements remain comparatively rare in publications, likely reflecting a detection bias rather than their true prevalence. The precise architecture of this deletion provides a meaningful contrast to the prevailing model for genomic disorders. Whereas many large deletions are mediated by recombination between flanking Alu repeats, the absence of such homology at our breakpoints suggests an alternative replication-based mechanism, such as microhomology-mediated end joining (10). This finding contributes to a more nuanced understanding of the mutational forces shaping the *AGK* gene.

In this case, CNV analysis proved essential for obtaining a definitive diagnosis, highlighting its value in the diagnostic workflow for autosomal recessive conditions when initial sequencing reveals only a single heterozygous pathogenic variant. It also illustrates the need for clinicians and geneticists to maintain a high index of suspicion when clinical presentation and initial genetic results are discordant. Finally, this case reaffirms that achieving a molecular diagnosis, even for an incurable condition like Sengers syndrome, is of paramount value. It provides families with clarity, ends an often protracted diagnostic odyssey, enables accurate genetic counseling, and guides multidisciplinary management toward optimizing supportive and palliative care. In summary, this case demonstrates how comprehensive genetic analysis, including CNV interrogation, can resolve diagnostically ambiguous cases and provide insights into mutational mechanisms.

## Patient perspective

The parents of the patient expressed relief at receiving a definitive diagnosis, despite its poor prognosis. They emphasized that the molecular confirmation ended a prolonged diagnostic odyssey and provided clarity for future family planning. They consented to the publication of this case in the hope that it would help other families facing similar diagnostic challenges.

## Data Availability

The original contributions presented in the study are included in the article/supplementary material, further inquiries can be directed to the corresponding author/s.
